# Plasma Insulin-Like Growth Factor-Binding Protein-2 Levels Predict Severe Septic Acute Kidney Injury: A Mendelian Randomization Analysis

**DOI:** 10.7759/cureus.82209

**Published:** 2025-04-13

**Authors:** Bozhi Zhao, Zuyi Zhao, Zhengkai Wang

**Affiliations:** 1 Intensive Care Unit, The First Affiliated Hospital of Xinjiang Medical University, Urumqi, CHN

**Keywords:** acute kidney injury, genome-wide association studies, insulin-like growth factor-binding protein, mendelian randomization, sepsis, sepsis-associated acute kidney injury

## Abstract

Background: Sepsis-associated acute kidney injury (SA-AKI) currently lacks highly sensitive biomarkers for early detection, resulting in delayed identification and intervention during its early stages and an independent risk of death.

Objective: This study aimed to investigate the relationship between insulin-like growth factor-binding protein-2 (IGFBP-2) levels and the occurrence of sepsis-induced kidney injury and to evaluate the causal relationship between the two through Mendelian randomization (MR) analysis.

Methods: This study employed a single-center, prospective cohort design involving 79 sepsis patients from the Intensive Care Unit (ICU) at the First Affiliated Hospital of Xinjiang Medical University, Ürümqi, China. The patients were divided into two groups, the SA-AKI group and the non-SA-AKI group, on the basis of whether they developed SA-AKI. The primary endpoint was whether SA-AKI occurred within 48 hours of admission. MR and sensitivity analyses were conducted to explore the causal relationships.

Results: The IGFBP-2 level had high diagnostic value for the prediction of SA-AKI. Receiver operating characteristic (ROC) curve analysis revealed that IGFBP-2 alone predicted SA-AKI, with an area under the curve (AUC) of 0.8994, a cut-off value of 709.004, a sensitivity of 88.64%, and a specificity of 85.71%. The combined prediction of the IGFBP-2 score, acute physiology and chronic health evaluation (APACHE) II score, sequential organ failure assessment (SOFA) score, and use of vasopressors had an AUC of 0.9604, a sensitivity of 93.18%, and a specificity of 82.86%. MR analysis revealed no causal relationship between genetically predicted IGFBP-2 levels and AKI (OR: 1.1507, 95% CI: 0.88-1.50, p = 0.2995).

Conclusion: Plasma IGFBP-2 levels can predict the occurrence of SA-AKI in sepsis patients. However, MR analysis suggests that there is no direct causal relationship between plasma IGFBP-2 levels and septic kidney injury, and the underlying mechanisms need to be further investigated in randomized controlled trials.

## Introduction

Sepsis is a systemic, harmful immune response to infection that can lead to severe acute organ dysfunction caused by infection [1]. Sepsis-associated acute kidney injury (SA-AKI) is a common and serious complication among critically ill patients, resulting in increased morbidity and mortality. The mortality rate of sepsis patients with acute kidney injury (AKI) is significantly greater than that of sepsis patients without AKI [2,3]. The systemic inflammatory response induced by sepsis leads to the release of many inflammatory mediators, which damage renal tubular epithelial cells and interstitial cells. Microcirculation dysfunction and insufficient tissue perfusion further aggravate renal hypoxia, inducing apoptosis and autophagy. Moreover, oxidative stress and mitochondrial dysfunction are also key factors in this pathological process [4]. The increased mortality rate is partly due to the unclear pathogenesis of SA-AKI, the lack of highly sensitive and specific biomarkers for early diagnosis, and the absence of effective specific treatments [5]. However, targeted studies on early inflammatory storms have not been successful. Most sepsis research has focused on the inflammatory process, with less attention given to anti-inflammatory pathways [6]. The insulin-like growth factor (IGF) regulatory pathway is evolutionarily conserved and regulates the growth of almost all organs in the body [7]. In this study, we investigated the ability of insulin-like growth factor-binding protein-2 (IGFBP-2) in the IGF pathway to predict kidney injury caused by sepsis, enabling early detection and intervention. Furthermore, IGFBP-2 has various functions independent of the IGF pathway. This protein can be transported into cells, bind to proteins such as p21, and enter the nucleus to regulate gene expression [8,9].

Previous studies have shown that IGFBP-2 acts as an oncogene and plays an important role in the study of various cancers, promoting processes such as cell proliferation, invasion, and migration [10]. The immune environment in tumors is crucial for tumor development, and IGFBP-2 has shown immunosuppressive effects in glioblastoma [11]. In pancreatic ductal adenocarcinoma cells, IGFBP-2 stimulates the expression of IL-10 [12]. These findings suggest that IGFBP-2 is also involved in immune processes. Some studies have shown that IGFBP-2 levels are correlated with disease severity and prognosis, and patients with higher plasma IGFBP-2 levels have a greater probability of requiring dialysis as the disease progresses [13]. Studies on male rats with acute kidney injury have shown high expression of IGFBP-2 in their renal tissues [14]. Additionally, patients with lupus nephritis, diabetic nephropathy, and chronic kidney disease have been shown to have elevated serum IGFBP-2 levels [8,15]. Therefore, this study aimed to explore the relationship between plasma IGFBP-2 levels and the occurrence of SA-AKI in patients and to investigate whether plasma IGFBP-2 levels can predict the development of SA-AKI.

This study aimed to explore whether plasma IGFBP-2 levels can predict the occurrence of septic kidney injury in critically ill patients by analysing the relationship between plasma IGFBP-2 levels and the development of sepsis-induced kidney injury within 48 hours.

## Materials and methods

Study design and participants

This study collected data from January 2024 to January 2025. Plasma samples were obtained from 80 sepsis patients admitted to the intensive care unit (ICU) of the First Affiliated Hospital of Xinjiang Medical University, Ürümqi, China. The patient population consisted of individuals of Asian ethnicity. The study was approved by the Ethics Committee of the First Affiliated Hospital of Xinjiang Medical University (Ethics approval number: K202309-12), and informed consent was obtained from the patients or their family members. Eligibility screening was conducted, and 80 adult sepsis patients with normal renal function who were admitted to the ICU within 24 hours due to sepsis were prospectively included. The development of acute kidney injury (AKI) was tracked over a three-day period in the ICU.

All participants met the diagnostic criteria for Sepsis-3 [16], which included a positive or suspected infection and a sequential organ failure assessment (SOFA) score of 2 or higher. The exclusion criteria included age <18 years, pregnancy or breastfeeding, preexisting kidney disease (e.g., nephrotic syndrome, lupus nephritis, interstitial nephritis, or end-stage renal disease), history of kidney transplantation, malignancy or hematologic diseases, obstructive urinary tract diseases, or a life expectancy of less than 48 hours.

Patients were divided into two groups on the basis of the presence of AKI: the sepsis non-AKI group and the sepsis AKI group. Sepsis patients without AKI were defined as those who did not develop AKI within the first seven days of sepsis, while sepsis AKI patients were defined as those who experienced a sudden and sustained decline in renal function within three days of admission, with an absolute increase in serum creatinine ≥0.3 mg/dL (or ≥26.5 µmol/L), a 1.5-fold increase in serum creatinine from baseline within the past seven days, or a urine output <0.5 mL/kg/h for six hours.

All patients underwent routine medical history recording in the ICU, acute physiology and chronic health evaluation (APACHE II) score, SOFA score, routine laboratory assessments, and plasma IGFBP-2 enzyme-linked immunosorbent assay (ELISA) on the day of ICU admission. Renal artery Doppler and abdominal ultrasound examinations were performed on the day of ICU admission.

Samples and laboratory analysis

Blood samples were obtained within 24 hours of participants enrolling in the study. Plasma was processed within 30 minutes of collection, and samples were stored at -80°C to avoid repeated freeze-thaw cycles. IGFBP-2 levels were determined using an ELISA. Serum samples were diluted 1:200 following the manufacturer's guidelines (Elabscience, Wuhan, China), and standard working solutions were prepared. Standards, blanks, and samples were placed in separate wells. Subsequently, 100 μL of either standard solution, blanks, or diluted serum samples was added to the appropriate wells and incubated at 37°C for 90 minutes. Following this, additional steps such as adding biotinylated antibody working solution, enzyme-conjugated working solution, substrate solution, and stop solution were carried out as per the manufacturer's instructions. Once the reaction was completed, the optical density (OD) of each well was read at 450 nm using a microplate reader.

Clinical data collection

All clinical data, including demographic data (age, sex, SOFA score, and APACHE II score), serum creatinine levels, routine blood parameters, length of stay, use of renal replacement therapy (RRT), and 28-day mortality rate, were extracted from the medical records.

MR analysis

Design of the Mendelian Randomization (MR) Method

MR analysis must satisfy three key assumptions. The first assumption is that genetic variants are significantly associated with exposure. The second assumption is that the genetic variation used as an instrumental variable (IV) for exposure is not correlated with other confounding factors. The third assumption is that the genetic variants affect the outcome only through the exposure and not through other pathways. Our MR analysis adhered rigorously to the principles outlined in the 2023 STROBE-MR Guidelines by Burgess et al. [17]. This study uses an MR design with a two-sample design to explore the causal relationship between serum IGFBP-2 and sepsis-induced kidney injury.

Exposure and Outcome Data Sources

Our data were obtained from publicly accessible genome-wide association study (GWAS) databases [18]. The exposure variable, IGFBP-2, was extracted from the IEU open GWAS project (https://gwas.mrcieu.ac.uk), specifically from study ID ebi-a-GCST90085740, which included a total of 400 participants and 5,188,525 single-nucleotide polymorphisms (SNPs). The outcome variable, tubular damage, was sourced from the FINNGEN database, encompassing a sample size of 340,209 individuals, with the study ID FinnGen R12 N14 DISIMPAIRRENTUB. FINNGEN is a significant GWAS resource that analyzes genomic and health data from around 500,000 participants in Finland [19]. Any samples with missing data will be omitted from this study.

IV Selection

We identified SNPs with a significance threshold of p < 10^-5^ to serve as IVs for the exposure [20]. These SNPs were confirmed to be independent, ensuring they were not in linkage disequilibrium (LD), with R^2^ < 0.001 and an LD distance greater than 10,000 kb. The extracted data for exposure and outcome were merged and harmonized to align the SNP effects on both the exposure and outcome with the same allele. Additionally, we calculated the R^2^ and F-statistics for the selected SNPs, with F-statistics above 10 indicating a strong association. The F-statistic was calculated using the formula: F = R^2^(N-K-1)/(1-R^2^), where R^2^ is the variance in exposure explained by the selected SNPs and N is the number of genetic samples for the phenotype.

MR and Sensitivity Analysis

The main analytical approach employed in this study was the standard inverse variance weighting (IVW) method. To further evaluate the causal relationship between the exposure and outcome, supplementary analyses were conducted using the MR-Egger, weighted median, and maximum likelihood methods. The MR analysis was carried out using R software (version 4.3.1; R Development Core Team, Vienna, Austria), incorporating two-sample MR (version 0.5.7), and MRPRESSO (version 1.0; https://github.com/rondolab/MR-PRESSO). A p-value of less than 0.05 was considered to indicate statistical significance.

To enhance the robustness of the results, we performed sensitivity analyses. We assessed heterogeneity using Cochran's Q test and evaluated horizontal pleiotropy through the MR-Egger intercept test. Scatter plots and funnel plots were utilized to visualize the results, aiding in the identification of outliers and pleiotropy. To ascertain the direction of causality and reduce bias from reverse causality, we employed the Steiger test [21].

Statistical analysis

Statistical analyses were performed via STATA 18 software (StataCorp LLC, College Station, TX) and Statistical Product and Service Solutions (SPSS, version 25; IBM SPSS Statistics for Windows, Armonk, NY). Data that were normally distributed are presented as the mean ± standard deviation (SD), whereas nonnormally distributed data are presented as the interquartile range (IQR). For normally distributed data, t-tests were used, whereas nonparametric tests were used for nonnormally distributed data. Simple linear regression was used for correlation analysis. Receiver operating characteristic (ROC) curve analysis was used to evaluate sensitivity and specificity. A p-value of < 0.05 was considered statistically significant.

## Results

Baseline characteristics of the study population

A total of 79 sepsis patients were included in this study, 35 of whom were in the non-AKI group and 44 in the AKI group. The 79 sepsis patients were divided into two groups on the basis of whether SA-AKI occurred: the SA-AKI group and the non-SA-AKI group. The baseline data for the two groups are shown in Table 1. No significant differences were observed between the two groups in terms of age, sex, or 28-day prognosis. In terms of routine examinations, no significant differences were found between the groups. However, the plasma IGFBP-2 concentrations differed significantly (p < 0.05). There was also a significant difference between the two groups in the use of vasopressors within 48 hours (p < 0.05), likely because patients using vasopressors required increased blood pressure to maintain organ perfusion, suggesting worse organ perfusion and a statistically significant probability of SA-AKI. Additionally, significant differences were observed in the APACHE II score and SOFA score (p < 0.05). The length of hospital stays also differed significantly between the two groups, but the 28-day survival rate did not significantly differ.

**Table 1:** Characteristics of the patients with sepsis at baseline. Abbreviations: AKI, Acute Kidney Injury; IGFBP-2, Insulin-Like Growth Factor-Binding Protein-2; Sequential Organ Failure Assessment (SOFA) Score, ranging from 0 to 24, with higher scores indicating more severe organ dysfunction; APACHE II, Acute Physiology and Chronic Health Evaluation II; p < 0.05, statistically significant Mean ± SD, Median (Q1, Q3) *The variable data follow a normal distribution using the t-test. **The variable data do not follow a normal distribution using the rank-sum test.

Variables	Without AKI (n = 35)	AKI (n = 44)	t/z	p-value
Gender (Male/Female)**	22/13	22/22	-1.136	0.2588
Age (years)**	59（49,69）	57.5（47,66.5）	0.716	0.3394
White Blood Cells*	15.48±0.81	17.33±1.14	-1.2603	0.2114
Neutrophil Percentage*	89.29±0.77	89.25±0.92	0.0288	0.9711
Platelets**	191（136,241）	189.6（127.5,234）	0.143	0.8076
Total Bilirubin*	32.34±6.76	36.03±8.19	-0.3361	0.7377
Creatinine*	85.79±10.38	109.43±8.60	-2.0305	0.0783
Bun*	18.43±4.26	21.66±2.53	-0.6559	0.5138
Use of Vasopressors(Y/N)**	14/21	35/9	-3.575	0.0002
Procalcitonin*	12.63±3.94	10.35±3.65	0.4229	0.6736
Interleukin 6*	708.16±266.79	733.48±217.95	-0.0742	0.9410
Lactate*	1.87±0.18	2.56±0.30	-1.8548	0.0674
Oxygenation Index*	273.81±13.64	251.61±11.73	1.2391	0.2191
IGFBP-2*	501.50±38.45	1393.62±116.51	-6.5976	＜0.0001
Length of Hospital Stay*	14.98±1.44	21.68±2.60	-2.1031	0.0387
SOFA Score*	6.60±0.50	8.77±0.49	-3.0817	0.0029
APACHE II Score*	16.40±0.72	19.93±0.89	-2.9865	0.0038
28-Day Survival Rate*	0.09±0.05	0.18±0.06	-1.2217	0.2255

Diagnostic predictive value of IGFBP-2 for SA-AKI

**Figure 1:**
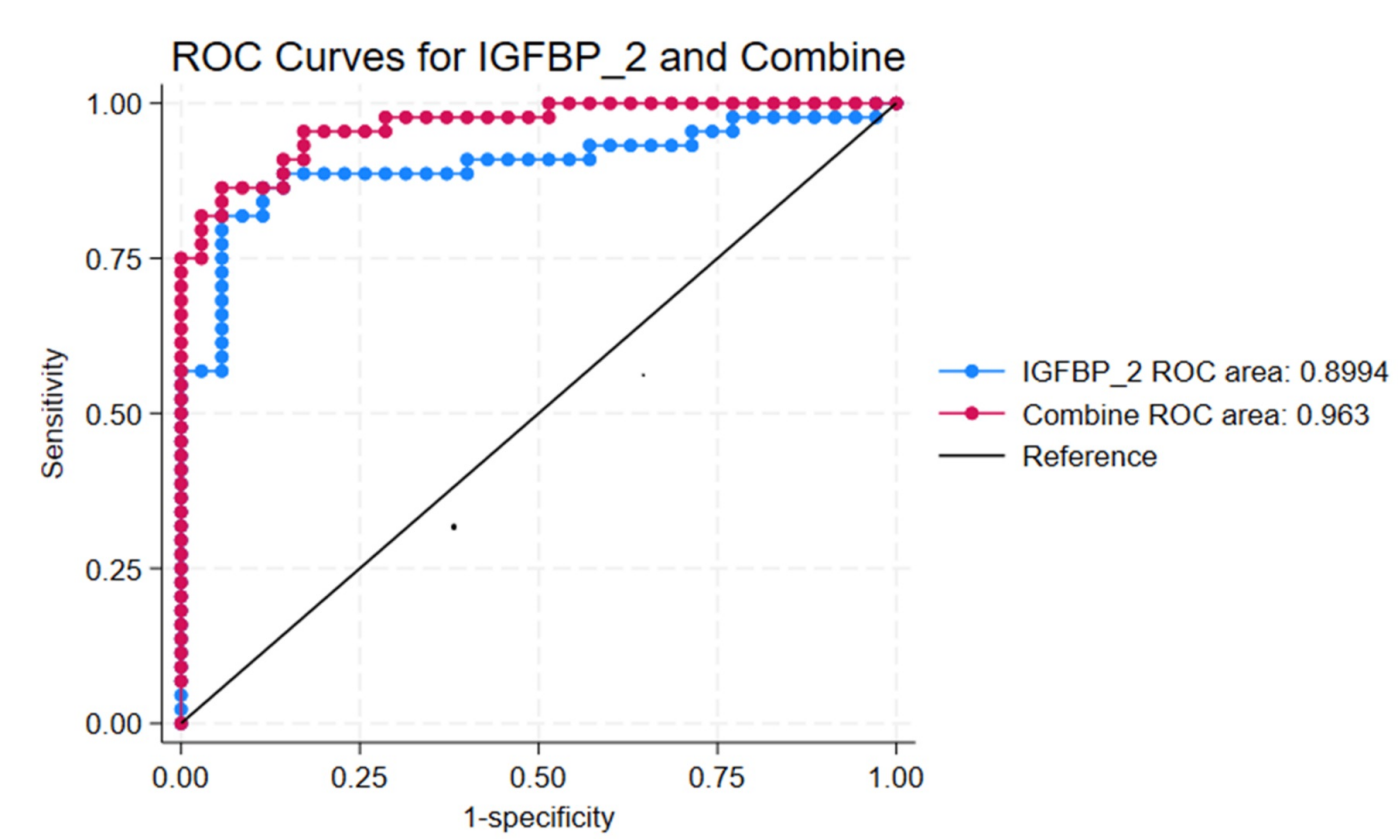
Correlation between plasma IGFBP-2 and septic kidney injury in sepsis patients and the ROC curve for IGFBP-2 in the prediction of SA-AKI. This study investigated the ROC curves for IGFBP-2 and the combined prediction of IGFBP-2 with the APACHE II score, SOFA score, and use of vasopressors for diagnosing SA-AKI and AKI in sepsis patients (sepsis AKI group, n = 35; SA-AKI group, n = 44; IGFBP-2, AUC = 0.8994; combined, AUC = 0.9604). Abbreviations: AKI, Acute Kidney Injury; IGFBP-2, Insulin-Like Growth Factor-Binding Protein-2; SA-AKI, Sepsis-Associated Acute Kidney Injury; Sequential Organ Failure Assessment (SOFA) Score, ranging from 0 to 24, with higher scores indicating more severe organ dysfunction; APACHE II, Acute Physiology and Chronic Health Evaluation II; ROC, Receiver Operating Characteristic

To evaluate the predictive value of IGFBP-2 for diagnosing SA-AKI, a ROC curve analysis was conducted (Figure 1). Univariate ROC curve analysis revealed that IGFBP-2 alone predicted SA-AKI with an AUC of 0.8994, a cut-off value of 709.004, a sensitivity of 88.64%, and a specificity of 85.71% (Table 2). We subsequently combined the statistically significant features from Table 1 for joint prediction, and the results revealed that combining the IGFBP-2 score with the APACHE II score, the SOFA score, and the use of vasopressors improved the prediction of SA-AKI, with an AUC of 0.9604, a sensitivity of 93.18%, and a specificity of 82.86% (Table 2).

**Table 2:** ROC values, p-values, sensitivity, specificity, and cut-off values for Figure 1. AUC, Area Under the Curve; IGFBP-2, Insulin-Like Growth Factor-Binding Protein-2; ROC, Receiver Operating Characteristic

Urinary Biomarkers	AUC	p value	Cut-off	Sensitivity (%)	Specificity (%)
IGFBP-2	0.8994	0.0374	709.004	88.64%	85.71%
Combine	0.9604	0.001	-0.279	93.18%	82.86%

MR analysis

The causal relationship between IGFBP-2 and tubular damage remains unclear. To explore this relationship, MR analysis was conducted. Following predefined screening criteria, 59 SNPs were selected to analyze the causal relationship between IGFBP-2 and tubular damage(Supplementary Table S1). Notably, all the statistical values exceeded 10, indicating that there was no weak instrumental variable bias.

The results from the IVW method, MR-Egger method, weighted median method, and maximum likelihood method were consistent. In the IVW analysis, no causal relationship was found between IGFBP-2 and tubular damage (OR: 1.1507, 95% CI: 0.88-1.50, p = 0.2995).

**Figure 2:**
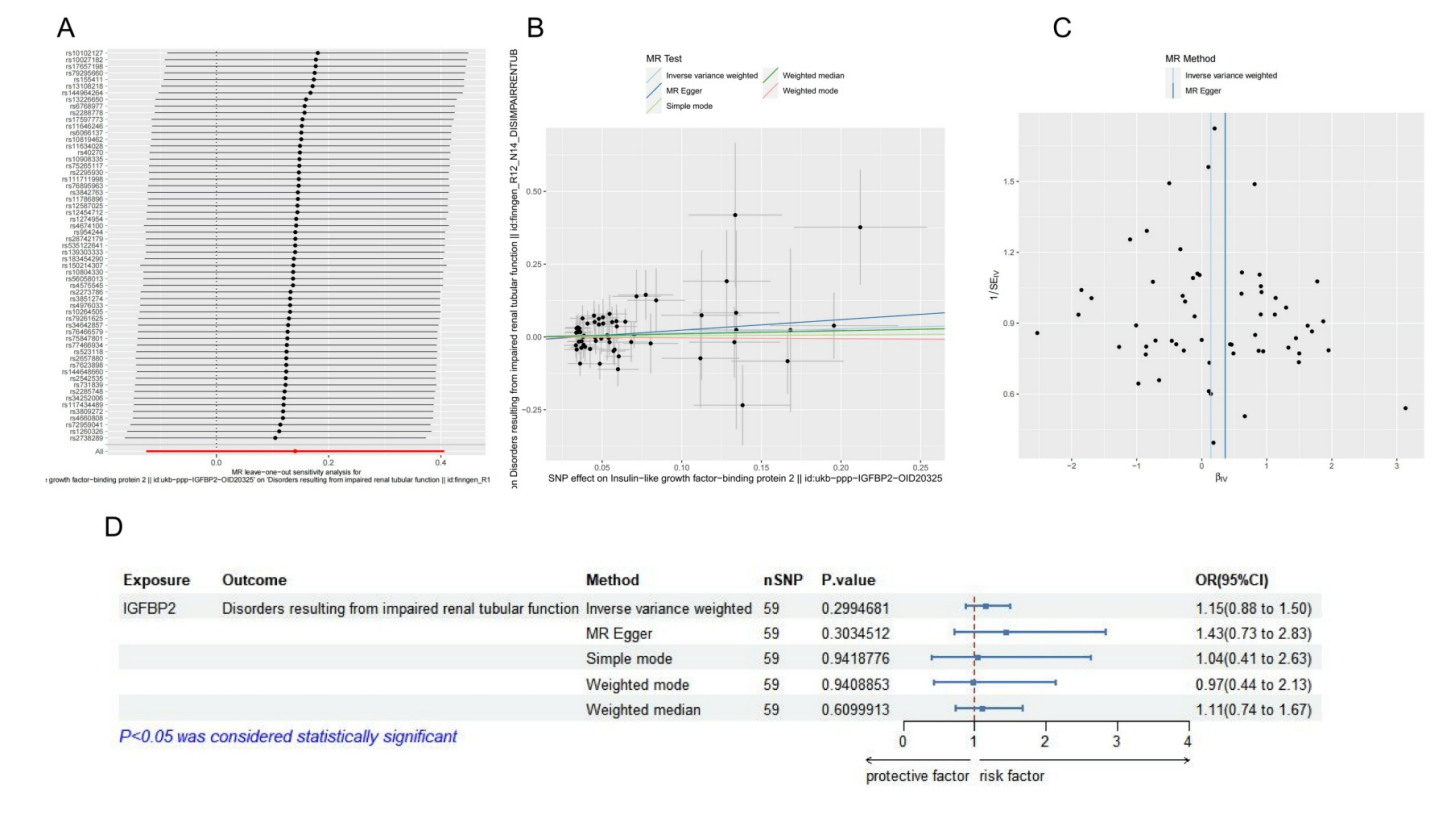
MR analysis correlation graph. (A) Forest plot of MR leave-one-out analysis for significant IVW estimates. (B) Scatter plot showing the impact of SNPs on IGFBP-2 and their effect on tubular damage. (C) Funnel plot for the correlation between IGFBP-2 and tubular damage. (D) Forest plot for MR analysis of the effect of IGFBP-2 on tubular damage, including sensitivity analysis, Steiger test, and F-statistic regression results. MR, Mendelian Randomization; SNP, Single-Nucleotide Polymorphism; IGFBP-2, Insulin-Like Growth Factor-Binding Protein-2; IVW, Inverse Variance Weighting

In the sensitivity analysis, Cochran's Q test revealed no significant heterogeneity (p < 0.05). Various methods, including MR-Egger regression, MR-PRESSO, and funnel plot analysis, revealed that horizontal pleiotropy did not significantly affect the occurrence of SA-AKI through pathways other than the exposure itself (p > 0.05). Furthermore, the leave-one-out analysis additionally ruled out any causal links between exposure driven by individual SNPs and the outcome. We also conducted a reverse MR analysis (Figure 2) using increased kidney injury as the exposure and IGFBP-2 levels as the outcome. The results were not statistically significant (Supplementary Table S2).

## Discussion

The risk of SA-AKI in sepsis patients is high and uneven over time. This necessitates the use of early biomarkers to provide early diagnosis and prognostic information [22]. Currently, serum creatinine (SCr) is commonly used in clinical practice to determine whether SA-AKI has occurred, but it is only a marker of renal insufficiency [23]. An increase in the SCr level is not directly related to renal tubular damage but reflects impaired filtration function, and many factors can lead to an increase in the SCr level [24]. During acute changes in kidney function, the SCr level does not immediately reflect the extent of kidney damage. SCr may not change until approximately 50% of kidney function is lost, and it reflects only renal function once it reaches a stable state, which may take several days [25]. This delay in changes makes early diagnosis and intervention difficult. In male acute kidney injury rat models, the IGFBP-2 protein is highly expressed in kidney tissues [14]. Moreover, patients with lupus nephritis, diabetic nephropathy, and chronic kidney disease have been shown to have elevated serum IGFBP-2 levels [5,12]. Therefore, IGFBP-2 is a potential new biomarker for predicting SA-AKI.

The mechanisms underlying SA-AKI include insufficient renal perfusion, renal vasoconstriction, inflammation, oxidative stress, and nephrotoxicity [26]. Renal Doppler ultrasound can detect impaired blood flow in large and microvascular vessels through these pathways [27]. We designed this study to detect serum IGFBP-2 levels as an early predictor of SA-AKI in critically ill sepsis patients. Our study revealed that sepsis patients with higher plasma IGFBP-2 levels have a significantly greater probability of developing SA-AKI than do those with lower IGFBP-2 levels. The diagnostic AUC of IGFBP-2 for SA-AKI in this study was 0.8994, with a cut-off value of 709.004, a sensitivity of 88.64%, and a specificity of 85.71%. When the APACHE II score, SOFA score, and use of vasopressors were combined, the prediction performance for SA-AKI improved, with an AUC of 0.9604, sensitivity of 93.18%, and specificity of 82.86%. These results indicate that IGFBP-2 is a promising biomarker for predicting SA-AKI. A meta-analysis has summarized the existing biomarkers for predicting SA-AKI, among which C-C motif chemokine ligand 14 (CCL14) and tissue inhibitor of metalloproteinase-2 and insulin-like growth factor-binding protein-7 (TIMP-2 and IGFBP-7) are currently the biomarkers with better predictive performance. The predictive performance was 0.8558 for CCL14 and 0.7563 for TIMP-2 and IGFBP-7 [28]. In this study, IGFBP-2 appeared to perform better; however, this may be due to the higher SOFA scores of the patients selected in this study, as the sample included more critically ill patients.

Our study demonstrated that measuring plasma IGFBP-2 levels in newly admitted patients can effectively predict the occurrence of SA-AKI, suggesting that IGFBP-2 plays a key role in the pathophysiology of SA-AKI. Previous studies have shown that plasma IGFBP-2 levels are positively correlated with dialysis requirements, disease severity, and mortality [13]. Currently, reliable laboratory biomarkers for the early diagnosis of septic kidney injury are still lacking. In these cases, IGFBP-2 may be useful in predicting the risk of severe septic kidney injury. Septicemia due to sepsis does not seem to alter plasma IGFBP-2 levels, and sheep injected with lipopolysaccharide maintained normal IGFBP-2 levels throughout the nine-hour observation period [26]. In humans, IGFBP-2 levels increase by 50% above baseline three hours after endotoxin administration and remain elevated for the next two hours [29]. Therefore, the increase in IGFBP-2 is more related to the progression of sepsis itself than to infection.

To explore the deeper causal relationship between IGFBP-2 and SA-AKI, we performed an MR analysis. The sensitivity analysis using four different MR methods revealed that plasma IGFBP-2 levels do not have a direct causal relationship with the occurrence of SA-AKI. The MR results suggest that plasma IGFBP-2 levels do not have a direct causal effect on septic kidney injury, which does not align with our initial hypothesis. However, as shown in Figure 2(B), IGFBP-2 is a risk factor for acute kidney injury rather than a protective factor, which is consistent with the trend observed in our previous studies [17]. Some studies have suggested that the severity of sepsis itself may influence the expression of plasma IGFBP-2. This result may be because more severe infections and septic shock increase the likelihood of SA-AKI. However, this does not affect the ability of plasma IGFBP-2 levels to predict the occurrence of SA-AKI. While there is no deeper causal relationship, plasma IGFBP-2 levels remain a good predictor of SA-AKI development on the basis of the results of this study. IGFBP-2 may have a complex mechanism in the process of SA-AKI in sepsis patients, but these mechanisms are not yet well understood. However, further studies are needed to clarify these mechanisms.

In this study, MR was performed using a strict genome-wide significance threshold (p < 10^-5^) for SNP selection [20]. The genetic sample libraries used in the study were selected from different populations to minimize confounding factors in genetic studies. Recent studies have shown that septic kidney injury may differ among various ethnic groups, highlighting the importance of diverse populations in research [30]. We also acknowledge other limitations. First， the GWAS sample size was relatively small, which reduced the power to detect causal biomarkers for proteins. In theory, with an increased sample size, more pathogenic protein biomarkers can be identified. Second, our study did not clarify the potential biological mechanisms linking these biomarkers to SA-AKI. Finally, although our study revealed that IGFBP-2 is a predictive biomarker, its clinical benefits (e.g., AUC, nomogram, and decision curve analysis) need external validation.

Our study also exhibits certain limitations as follows:

Lack of direct reflection of renal IGFBP-2: We measured IGFBP-2 levels in the blood, but the kidney may not be the sole source of IGFBP-2. Patients may develop oliguria or anuria after admission, making it difficult to consistently collect urine samples, which prevents urine from being used as the primary study specimen. Renal tissue biopsy, being an invasive procedure, could cause unnecessary harm to patients and was therefore not included in the study protocol.

Single-center design and limited sample size: The single-center design and limited sample size of this study may have introduced potential bias, particularly considering the presence of various comorbidities among the enrolled patients. This may limit the generalizability of our findings. We emphasize the need for larger sample sizes and multi-center studies to validate our results.

Representativeness of population ancestry: Our study faced challenges regarding the representativeness of population ancestry. The MR analysis primarily involved individuals of European ancestry, while the cohort study focused on an Asian population. We recommend further validation in multi-center studies involving diverse ancestral populations to enhance the broader applicability of our conclusions.

## Conclusions

IGFBP-2 levels can predict the occurrence of SA-AKI in sepsis patients. However, the results of MR suggest that there is no direct causal relationship between plasma IGFBP-2 levels and septic kidney injury. These results also emphasize the need for further pathophysiological studies and randomized controlled trials to confirm these observations.

## Appendices

**Table 3:** Supplementary Table S1: 59 SNPs selected to analyze the causal relationship between IGFBP-2 and tubular damage. IGFBP-2, Insulin-Like Growth Factor-Binding Protein-2; SNPs, Single-Nucleotide Polymorphisms

SNP	effect_allele.exposure	other_allele.exposure	effect_allele.outcome	other_allele.outcome	beta.exposure	beta.outcome	eaf.exposure	eaf.outcome	remove	palindromic	ambiguous	chr	pos	se.outcome	samplesize.outcome	pval.outcome	mr_keep.outcome	chr.exposure	pos.exposure	se.exposure	pval.exposure	samplesize.exposure	mr_keep.exposure	pval_origin.exposure	data_source.exposure	action	mr_keep	R2	F	F_pow_beta_se
rs10027182	A	G	A	G	0.0604521	-0.0666254	0.743739	0.755225	FALSE	FALSE	FALSE	4	148060255	0.0481892	496697	0.166794	TRUE	4	148060255	0.00844464	8.1489189821385e-13	34028	TRUE	reported	MedicineITLab	2	TRUE	0.00139301518912345	47.4648540878084	51.2460627496128
rs10102127	C	T	C	T	-0.0599107	0.110839	0.908517	0.843077	FALSE	FALSE	FALSE	8	132343095	0.0575801	496697	0.054235	TRUE	8	132343095	0.0127589	2.65803074470695e-06	34028	TRUE	reported	MedicineITLab	2	TRUE	0.000596639826437744	20.3133865097722	22.0486661022269
rs10264505	A	C	A	C	0.0592088	0.0361555	0.0907565	0.151695	FALSE	FALSE	FALSE	7	99540251	0.0577728	496697	0.531432	TRUE	7	99540251	0.0128259	3.90562012567539e-06	34028	TRUE	reported	MedicineITLab	2	TRUE	0.00057857605805834	19.6980257575874	21.310656034313
rs10804330	C	T	C	T	0.0337226	0.0147243	0.432451	0.432877	FALSE	FALSE	FALSE	2	226321033	0.0416	496697	0.723376	TRUE	2	226321033	0.00747561	6.45163813025318e-06	34028	TRUE	reported	MedicineITLab	2	TRUE	0.000558228964277377	19.0049078285153	20.3492697937992
rs10819462	T	C	T	C	-0.0383274	0.0272573	0.692053	0.727947	FALSE	FALSE	FALSE	9	129100451	0.046376	496697	0.556702	TRUE	9	129100451	0.00798167	1.57148413162342e-06	34028	TRUE	reported	MedicineITLab	2	TRUE	0.000626129328827361	21.3180243829786	23.0585069103798
rs10908335	G	A	G	A	-0.0353704	0.0162884	0.580711	0.640846	FALSE	FALSE	FALSE	1	37273951	0.0428624	496697	0.703934	TRUE	1	37273951	0.00746051	2.1264736227575e-06	34028	TRUE	reported	MedicineITLab	2	TRUE	0.000609233056336264	20.7424009312128	22.477236171614
rs111711998	A	G	A	G	0.111691	-0.0733426	0.023484	0.0147518	FALSE	FALSE	FALSE	16	7289577	0.169584	496697	0.66539	TRUE	16	7289577	0.0242079	3.95302899633532e-06	34028	TRUE	reported	MedicineITLab	2	TRUE	0.000572160390908445	19.4794748449927	21.2873757111501
rs11634028	A	T	A	T	0.0424477	-0.0413786	0.221664	0.110504	FALSE	TRUE	FALSE	15	75983809	0.0658718	496697	0.529893	TRUE	15	75983809	0.00892385	1.96829414227275e-06	34028	TRUE	reported	MedicineITLab	2	TRUE	0.0006217282572261	21.16808648	22.6257926709688
rs11646246	A	G	A	G	0.0334942	-0.0286195	0.514145	0.584805	FALSE	FALSE	FALSE	16	56545685	0.0418202	496697	0.493756	TRUE	16	56545685	0.00736326	5.39460934038725e-06	34028	TRUE	reported	MedicineITLab	2	TRUE	0.000560481790448899	19.0816483182283	20.6918321465896
rs117434489	T	C	T	C	-0.0715876	-0.139537	0.0647288	0.0524994	FALSE	FALSE	FALSE	11	105727363	0.0911853	496697	0.125953	TRUE	11	105727363	0.0150788	2.05878028135561e-06	34028	TRUE	reported	MedicineITLab	2	TRUE	0.000620498474639718	21.1261898666783	22.5393840573136
rs11786896	T	C	T	C	0.0804958	-0.022233	0.0493565	0.0422444	FALSE	FALSE	FALSE	8	143944186	0.102663	496697	0.828549	TRUE	8	143944186	0.0171894	2.82885052391069e-06	34028	TRUE	reported	MedicineITLab	2	TRUE	0.000608048856040225	20.7020582384543	21.9293119861585
rs12454712	C	T	C	T	0.045565	-0.00154022	0.378086	0.488364	FALSE	FALSE	FALSE	18	63178651	0.0412848	496697	0.97024	TRUE	18	63178651	0.00760623	2.09223276983561e-09	34028	TRUE	reported	MedicineITLab	2	TRUE	0.00097636830896954	33.2543766004448	35.8858999477614
rs12587025	G	A	G	A	0.0494467	-0.00544799	0.133489	0.186188	FALSE	FALSE	FALSE	14	50691761	0.0532423	496697	0.918499	TRUE	14	50691761	0.0109033	5.75956919203823e-06	34028	TRUE	reported	MedicineITLab	2	TRUE	0.000565619249286744	19.256652509567	20.566420071221
rs1260326	C	T	C	T	0.064544	0.0524695	0.608381	0.650245	FALSE	FALSE	FALSE	2	27508073	0.043362	496697	0.226266	TRUE	2	27508073	0.00754931	1.23452531517069e-17	34028	TRUE	reported	MedicineITLab	2	TRUE	0.00198509431323762	67.6791686350053	73.0966097061573
rs1274954	G	A	G	A	0.0379607	-1.77E-05	0.258561	0.282935	FALSE	FALSE	FALSE	14	68830435	0.0457866	496697	0.999692	TRUE	14	68830435	0.00839399	6.11504982517029e-06	34028	TRUE	reported	MedicineITLab	2	TRUE	0.000552505830392138	18.8099559952797	20.4517996467643
rs13108218	G	A	G	A	-0.057154	0.0483036	0.618883	0.678074	FALSE	FALSE	FALSE	4	3442204	0.0442845	496697	0.27538	TRUE	4	3442204	0.00762743	6.72047546628297e-14	34028	TRUE	reported	MedicineITLab	2	TRUE	0.00154095562020857	52.5134768705372	56.1483243071733
rs13226650	G	A	G	A	0.0578442	-0.0434386	0.196543	0.17717	FALSE	FALSE	FALSE	7	73602675	0.0538022	496697	0.41945	TRUE	7	73602675	0.009241	3.86180197218962e-10	34028	TRUE	reported	MedicineITLab	2	TRUE	0.00105674415254991	35.9948138437146	39.1815576834258
rs139303333	A	G	A	G	0.134251	0.024169	0.017788	0.00345828	FALSE	FALSE	FALSE	16	2098137	0.340722	496697	0.94345	TRUE	16	2098137	0.0300609	7.97021432398605e-06	34028	TRUE	reported	MedicineITLab	2	TRUE	0.00062979239324615	21.4428204978328	19.9448649917837
rs144648660	A	G	A	G	-0.133573	-0.41854	0.0176489	0.00748449	FALSE	FALSE	FALSE	20	14548771	0.24728	496697	0.090536	TRUE	20	14548771	0.0293599	5.37761863396381e-06	34028	TRUE	reported	MedicineITLab	2	TRUE	0.000618659568277067	21.0635416317678	20.697992072713
rs144964264	G	A	G	A	0.16647	-0.0831339	0.0122293	0.0385218	FALSE	FALSE	FALSE	8	13512143	0.11156	496697	0.456153	TRUE	8	13512143	0.0351556	2.18806389526738e-06	34028	TRUE	reported	MedicineITLab	2	TRUE	0.000669514046943505	22.7961473021347	22.422443079297
rs150214307	A	G	A	G	0.195576	0.0387365	0.00983034	0.0366909	FALSE	FALSE	FALSE	3	153172482	0.11345	496697	0.732771	TRUE	3	153172482	0.0402605	1.18716203614496e-06	34028	TRUE	reported	MedicineITLab	2	TRUE	0.00074462783833641	25.3555873033982	23.5978692645124
rs155411	C	T	C	T	0.0485548	-0.0920503	0.144741	0.197641	FALSE	FALSE	FALSE	3	1313313	0.0518675	496697	0.075943	TRUE	3	1313313	0.0105654	4.31429661247571e-06	34028	TRUE	reported	MedicineITLab	2	TRUE	0.000583691752246823	19.8722948565564	21.1199288834149
rs17597773	G	C	G	C	0.0546452	-0.0179674	0.246511	0.301188	FALSE	TRUE	FALSE	1	220881419	0.0450543	496697	0.690044	TRUE	1	220881419	0.00858256	1.9274805308006e-10	34028	TRUE	reported	MedicineITLab	2	TRUE	0.00110929551036438	37.7868057696496	40.5387490604611
rs17657198	C	A	C	A	0.0361462	-0.0914121	0.489728	0.424983	FALSE	FALSE	FALSE	17	54023616	0.0421135	496697	0.029961	TRUE	17	54023616	0.00757796	1.84297735926021e-06	34028	TRUE	reported	MedicineITLab	2	TRUE	0.000652998169098065	22.2334340934866	22.7520575425439
rs183454290	G	A	G	A	0.112449	0.0743674	0.0273863	0.00881724	FALSE	FALSE	FALSE	5	34372483	0.222191	496697	0.737852	TRUE	5	34372483	0.0253077	8.86012808010486e-06	34028	TRUE	reported	MedicineITLab	2	TRUE	0.000673619940804946	22.9360422812729	19.7426678938781
rs2273786	A	G	A	G	-0.0339392	-0.0296738	0.369372	0.341536	FALSE	FALSE	FALSE	9	111586233	0.0433393	496697	0.493542	TRUE	9	111586233	0.00759698	7.91534775450494e-06	34028	TRUE	reported	MedicineITLab	2	TRUE	0.000536624403098416	18.2689855232783	19.9581938286496
rs2285748	G	A	G	A	-0.0453115	-0.0514811	0.195721	0.30748	FALSE	FALSE	FALSE	17	5391681	0.0450042	496697	0.252657	TRUE	17	5391681	0.00928216	1.05244643173405e-06	34028	TRUE	reported	MedicineITLab	2	TRUE	0.000646384642918678	22.0081095639928	23.8297111590742
rs2288778	A	G	A	G	0.0340061	-0.0432075	0.354737	0.439923	FALSE	FALSE	FALSE	5	151541005	0.0425295	496697	0.309657	TRUE	5	151541005	0.00767761	9.45583749597545e-06	34028	TRUE	reported	MedicineITLab	2	TRUE	0.000529403615204936	18.0230288676026	19.6183085310492
rs2295930	T	C	T	C	0.0371339	-0.014556	0.290642	0.285723	FALSE	FALSE	FALSE	9	98872229	0.0457919	496697	0.750582	TRUE	9	98872229	0.00813166	4.95746891827398e-06	34028	TRUE	reported	MedicineITLab	2	TRUE	0.000568584435539602	19.3576604681211	20.8536799389708
rs2542535	G	T	G	T	0.0546687	0.0791753	0.127685	0.113823	FALSE	FALSE	FALSE	2	71273862	0.0653571	496697	0.225732	TRUE	2	71273862	0.011004	6.76191955875189e-07	34028	TRUE	reported	MedicineITLab	2	TRUE	0.000665764617114598	22.6683986797089	24.6817716436117
rs2657880	C	G	C	G	0.0562678	0.0511532	0.181447	0.181708	FALSE	TRUE	FALSE	12	56469986	0.0532683	496697	0.336907	TRUE	12	56469986	0.0095332	3.58467675784435e-09	34028	TRUE	reported	MedicineITLab	2	TRUE	0.000940473282796296	32.0306679078242	34.8371374146537
rs2738289	A	T	A	T	0.212076	0.376644	0.00789073	0.0112078	FALSE	TRUE	FALSE	2	216479299	0.196961	496697	0.055841	TRUE	2	216479299	0.0416829	3.62167937614954e-07	34028	TRUE	reported	MedicineITLab	2	TRUE	0.000704189805407178	23.9776471334537	25.8861339461965
rs28742179	G	A	G	A	-0.0531634	-0.00580349	0.114743	0.0603864	FALSE	FALSE	FALSE	15	98664810	0.0868435	496697	0.946719	TRUE	15	98664810	0.0115887	4.48507799729482e-06	34028	TRUE	reported	MedicineITLab	2	TRUE	0.000574183967105575	19.5484080472174	21.0453452734598
rs34252006	C	T	C	T	-0.047952	-0.0621818	0.144491	0.225278	FALSE	FALSE	FALSE	17	2967227	0.0496395	496697	0.210327	TRUE	17	2967227	0.0106704	6.99165515948176e-06	34028	TRUE	reported	MedicineITLab	2	TRUE	0.000568471670003004	19.3538191414105	20.1953810093593
rs34642857	C	T	C	T	-0.0506604	-0.0459581	0.2531	0.177104	FALSE	FALSE	FALSE	3	123332172	0.0540655	496697	0.395299	TRUE	3	123332172	0.00850519	2.57833889427641e-09	34028	TRUE	reported	MedicineITLab	2	TRUE	0.000970335296386113	33.0486971121414	35.4788218138446
rs3809272	A	G	A	G	-0.037703	-0.0638806	0.304602	0.354882	FALSE	FALSE	FALSE	12	111362454	0.0435728	496697	0.14263	TRUE	12	111362454	0.0079913	2.38166130109656e-06	34028	TRUE	reported	MedicineITLab	2	TRUE	0.000602210050965921	20.5031463950019	22.2595790245732
rs3842763	T	G	T	G	0.0541306	-0.00357616	0.235871	0.237414	FALSE	FALSE	FALSE	11	2157974	0.0487795	496697	0.941557	TRUE	11	2157974	0.00879378	7.48135046825707e-10	34028	TRUE	reported	MedicineITLab	2	TRUE	0.00105622613193819	35.9771503717041	37.8908623308917
rs3851274	A	G	A	G	-0.0347452	-0.0328298	0.640557	0.685813	FALSE	FALSE	FALSE	1	96519338	0.0444856	496697	0.460522	TRUE	1	96519338	0.00767136	5.92038568681715e-06	34028	TRUE	reported	MedicineITLab	2	TRUE	0.000555913779808025	18.926043520138	20.5137427215472
rs40270	C	A	C	A	-0.0460026	0.0135194	0.77381	0.706441	FALSE	FALSE	FALSE	5	56508725	0.0452935	496697	0.765332	TRUE	5	56508725	0.0087624	1.52096773059174e-07	34028	TRUE	reported	MedicineITLab	2	TRUE	0.000740802586866518	25.2252357406111	27.5625
rs4575545	A	G	A	G	0.0362521	0.0166343	0.305627	0.304724	FALSE	FALSE	FALSE	16	79721549	0.0447916	496697	0.710362	TRUE	16	79721549	0.0080349	6.4271731486167e-06	34028	TRUE	reported	MedicineITLab	2	TRUE	0.000557803041688047	18.9903991989135	20.3566067290924
rs4660808	T	C	T	C	-0.0448962	-0.0730769	0.226768	0.214303	FALSE	FALSE	FALSE	1	39552837	0.0504827	496697	0.147739	TRUE	1	39552837	0.00880759	3.44262723356032e-07	34028	TRUE	reported	MedicineITLab	2	TRUE	0.000706871956466819	24.0690389193712	25.9839390372197
rs4674100	A	G	A	G	-0.0700512	-0.00714468	0.292765	0.313162	FALSE	FALSE	FALSE	2	216615701	0.0448796	496697	0.873514	TRUE	2	216615701	0.00858953	3.47936507649094e-16	34028	TRUE	reported	MedicineITLab	2	TRUE	0.00203209522354732	69.2848655207099	66.5108283731732
rs4976033	G	A	G	A	-0.035695	-0.0293213	0.40131	0.403257	FALSE	FALSE	FALSE	5	68418419	0.0419797	496697	0.484888	TRUE	5	68418419	0.00759491	2.60345454790239e-06	34028	TRUE	reported	MedicineITLab	2	TRUE	0.000612247124625732	20.8450830046473	22.0886670484212
rs523118	G	T	G	T	-0.0589621	-0.0541505	0.766673	0.848881	FALSE	FALSE	FALSE	3	136247046	0.0571567	496697	0.343433	TRUE	3	136247046	0.00875026	1.60213829043185e-11	34028	TRUE	reported	MedicineITLab	2	TRUE	0.00124380141841695	42.3742922678824	45.4050304059073
rs535122641	C	G	C	G	0.168269	0.0235464	0.0147605	0.00582565	FALSE	TRUE	FALSE	2	216633326	0.280138	496697	0.933014	TRUE	2	216633326	0.0358692	2.71625163034297e-06	34028	TRUE	reported	MedicineITLab	2	TRUE	0.000823533191359948	28.0446360578468	22.0072028404004
rs56058013	T	C	T	C	0.0347492	0.0169147	0.419136	0.308311	FALSE	FALSE	FALSE	14	104977675	0.0450214	496697	0.707138	TRUE	14	104977675	0.00759179	4.71226819491523e-06	34028	TRUE	reported	MedicineITLab	2	TRUE	0.000587961707685776	20.0177547389766	20.950830757271
rs6066137	T	C	T	C	0.0392783	-0.0338105	0.264093	0.204227	FALSE	FALSE	FALSE	20	46962019	0.0511783	496697	0.508842	TRUE	20	46962019	0.00835175	2.56353941969905e-06	34028	TRUE	reported	MedicineITLab	2	TRUE	0.000599673952815198	20.416749311253	22.1182312430684
rs6768977	G	A	G	A	0.036942	-0.0372724	0.533429	0.52191	FALSE	FALSE	FALSE	3	12466670	0.0414784	496697	0.368867	TRUE	3	12466670	0.00741686	6.33169519573368e-07	34028	TRUE	reported	MedicineITLab	2	TRUE	0.000679305557448399	23.1297630743381	24.8085079359972
rs72959041	A	G	A	G	-0.0773928	-0.144587	0.0497049	0.064811	FALSE	FALSE	FALSE	6	127133748	0.0852558	496697	0.089902	TRUE	6	127133748	0.0172435	7.18124923883475e-06	34028	TRUE	reported	MedicineITLab	2	TRUE	0.000565833698627749	19.2639576259015	20.1442116537212
rs731839	A	G	A	G	0.0479991	0.0427056	0.667465	0.656955	FALSE	FALSE	FALSE	19	33408159	0.043426	496697	0.325405	TRUE	19	33408159	0.00781856	8.29793445434079e-10	34028	TRUE	reported	MedicineITLab	2	TRUE	0.0010227324696089	34.8351220212864	37.6888288176023
rs75265117	G	C	G	C	0.0682564	-0.0174342	0.118493	0.0999837	FALSE	TRUE	FALSE	2	164662289	0.0688204	496697	0.800014	TRUE	2	164662289	0.0114016	2.14333472405272e-09	34028	TRUE	reported	MedicineITLab	2	TRUE	0.000973274206144295	33.1488910989347	35.8389400175475
rs75847801	T	A	T	A	-0.0505149	-0.0673053	0.192489	0.124369	FALSE	TRUE	FALSE	19	7248655	0.0634025	496697	0.288438	TRUE	19	7248655	0.0100323	4.77276443701278e-07	34028	TRUE	reported	MedicineITLab	2	TRUE	0.000793274244629559	27.0133784651622	25.3535030796432
rs7623898	T	C	T	C	0.0408105	0.0458422	0.330128	0.342584	FALSE	FALSE	FALSE	3	46932682	0.0435767	496697	0.292805	TRUE	3	46932682	0.00782511	1.83484760775554e-07	34028	TRUE	reported	MedicineITLab	2	TRUE	0.000736627643988615	25.0829690227321	27.1996257716644
rs76466579	T	C	T	C	0.128222	0.191398	0.0185049	0.0140308	FALSE	FALSE	FALSE	20	57401390	0.174494	496697	0.272696	TRUE	20	57401390	0.0273998	2.87335972436047e-06	34028	TRUE	reported	MedicineITLab	2	TRUE	0.000597213982652641	20.3329460934543	21.8993037715027
rs76895963	G	T	G	T	0.133041	-0.0179274	0.020778	0.030216	FALSE	FALSE	FALSE	12	4275678	0.121931	496697	0.883109	TRUE	12	4275678	0.0284608	2.94604923100582e-06	34028	TRUE	reported	MedicineITLab	2	TRUE	0.000720254368250929	24.5250394008661	21.851275584638
rs77466934	A	G	A	G	-0.0839268	-0.125794	0.0437434	0.0371896	FALSE	FALSE	FALSE	11	54780575	0.108716	496697	0.247237	TRUE	11	54780575	0.0180213	3.20730307022974e-06	34028	TRUE	reported	MedicineITLab	2	TRUE	0.000589275393010392	20.0625068641888	21.6884789898289
rs79261625	G	T	G	T	-0.134133	-0.0828337	0.0187346	0.0306959	FALSE	FALSE	FALSE	17	56481262	0.120328	496697	0.491202	TRUE	17	56481262	0.027124	7.6078159936829e-07	34028	TRUE	reported	MedicineITLab	2	TRUE	0.000661503554867847	22.5232191474665	24.4547825083515
rs79295660	T	C	T	C	-0.138209	0.234328	0.0147379	0.0237566	FALSE	FALSE	FALSE	1	69880031	0.13739	496697	0.088089	TRUE	1	69880031	0.0305898	6.23878472271997e-06	34028	TRUE	reported	MedicineITLab	2	TRUE	0.000554740696652448	18.8860838235935	20.413589363273
rs954244	G	C	G	C	-0.0385888	-0.00455719	0.252909	0.191121	FALSE	TRUE	FALSE	2	120551655	0.0526791	496697	0.931062	TRUE	2	120551655	0.00845092	4.96592321450336e-06	34028	TRUE	reported	MedicineITLab	2	TRUE	0.000562717383518278	19.1578021198758	20.8504083980595

**Table 4:** Supplementary Table S2: Results of the Mendelian randomization (MR) analysis were not statistically significant.

id.exposure	id.outcome	outcome	exposure	method	nsnp	b	se	pval
finngen_R12_N14_DISIMPAIRRENTUB	ukb-ppp-IGFBP2-OID20325	Insulin-like growth factor-binding protein 2 || id:ukb-ppp-IGFBP2-OID20325	Disorders resulting from impaired renal tubular function || id:finngen_R12_N14_DISIMPAIRRENTUB	Inverse variance weighted	18	0.000663444350718056	0.00921172540368102	0.94258461896204
finngen_R12_N14_DISIMPAIRRENTUB	ukb-ppp-IGFBP2-OID20325	Insulin-like growth factor-binding protein 2 || id:ukb-ppp-IGFBP2-OID20325	Disorders resulting from impaired renal tubular function || id:finngen_R12_N14_DISIMPAIRRENTUB	MR Egger	18	0.0114910155448035	0.0209352522502982	0.590663579948712
finngen_R12_N14_DISIMPAIRRENTUB	ukb-ppp-IGFBP2-OID20325	Insulin-like growth factor-binding protein 2 || id:ukb-ppp-IGFBP2-OID20325	Disorders resulting from impaired renal tubular function || id:finngen_R12_N14_DISIMPAIRRENTUB	Simple mode	18	0.0231094450649843	0.0229612974297346	0.328309748265805
finngen_R12_N14_DISIMPAIRRENTUB	ukb-ppp-IGFBP2-OID20325	Insulin-like growth factor-binding protein 2 || id:ukb-ppp-IGFBP2-OID20325	Disorders resulting from impaired renal tubular function || id:finngen_R12_N14_DISIMPAIRRENTUB	Weighted mode	18	-0.01738	0.0229080700582115	0.458483680192798
finngen_R12_N14_DISIMPAIRRENTUB	ukb-ppp-IGFBP2-OID20325	Insulin-like growth factor-binding protein 2 || id:ukb-ppp-IGFBP2-OID20325	Disorders resulting from impaired renal tubular function || id:finngen_R12_N14_DISIMPAIRRENTUB	Weighted median	18	-0.00382	0.0128888366774881	0.76699384580648
